# Collagen-Hydroxyapatite Scaffolds Induce Human Adipose Derived Stem Cells Osteogenic Differentiation *In Vitro*

**DOI:** 10.1371/journal.pone.0151181

**Published:** 2016-03-16

**Authors:** Giovanna Calabrese, Raffaella Giuffrida, Claudia Fabbi, Elisa Figallo, Debora Lo Furno, Rosario Gulino, Cristina Colarossi, Francesco Fullone, Rosario Giuffrida, Rosalba Parenti, Lorenzo Memeo, Stefano Forte

**Affiliations:** 1 IOM Ricerca, Viagrande, Italy; 2 Finceramica, Faenza, Italy; 3 Department of Biomedical and Biotechnological Sciences, Physiology Section, University of Catania, Catania, Italy; 4 Department of Experimental Oncology, Mediterranean Institute of Oncology, Viagrande, Italy; University of Catania, ITALY

## Abstract

Mesenchymal stem cells (MSCs) play a crucial role in regulating normal skeletal homeostasis and, in case of injury, in bone healing and reestablishment of skeletal integrity. Recent scientific literature is focused on the development of bone regeneration models where MSCs are combined with biomimetic three-dimensional scaffolds able to direct MSC osteogenesis. In this work the osteogenic potential of human MSCs isolated from adipose tissue (hADSCs) has been evaluated *in vitro* in combination with collagen/Mg doped hydroxyapatite scaffolds. Results demonstrate the high osteogenic potential of hADSCs when cultured in specific differentiation induction medium, as revealed by the Alizarin Red S staining and gene expression profile analysis. In combination with collagen/hydroxyapatite scaffold, hADSCs differentiate into mature osteoblasts even in the absence of specific inducing factors; nevertheless, the supplement of the factors markedly accelerates the osteogenic process, as confirmed by the expression of specific markers of pre-osteoblast and mature osteoblast stages, such as osterix, osteopontin (also known as bone sialoprotein I), osteocalcin and specific markers of extracellular matrix maturation and mineralization stages, such as ALPL and osteonectin. Hence, the present work demonstrates that the scaffold *per se* is able to induce hADSCs differentiation, while the addition of osteo-inductive factors produces a significant acceleration of the osteogenic process. This observation makes the use of our model potentially interesting in the field of regenerative medicine for the treatment of bone defects.

## Introduction

MSCs are a group of clonogenic cells capable of multilineage differentiation into a range of specialised cell mesoderm-types such as osteoblasts, chondrocytes and adipocytes [1–3] and into other non-mesoderm types such as neuronal cells and hepatocyte [4, 5]. Traditionally, hMSC have been isolated from bone marrow, but these are also located in other tissues of the human body, such as adipose tissue [6], umbilical cord blood, chorionic villi of the placenta [7], amniotic fluid [8], peripheral blood [9], fetal liver [10], lung [11] and even in exfoliated deciduous teeth [12].

In recent years, a general interest in therapies based on the use of MSCs as treatment for bone repair is emerging. Indeed, MSCs isolated from bone marrow (bmMSCs) have been already used in clinical trials for bone damage treatment [13, 14].

Autologous bone grafting is so far the most commonly used method for the treatment of bone defects. However, the limitations of this approach, including morbidity at the harvesting site and limited availability, lead research towards the development of alternative bone substitutes of both biological and synthetic origin. Recent literature is rich of articles concerning the use of different types of scaffolds as fundamental tools to use in regenerative medicine [15–17]. These scaffolds are named ‘biomimetic’, because they have been developed to mimic the biochemical and biophysical properties of osteochondral structure layers [18].

Scaffolds offer a three-dimensional environment that is necessary for the production of cartilaginous matrix. A variety of scaffolds have been evaluated for MSC adhesion, proliferation, migration and differentiation. Scaffolds provide mechanical support while MSCs proliferate and eventually differentiate into functional tissue-specific cells [19].

Indeed, scaffolds loaded with MSCs should facilitate bone regeneration by supporting cell colonization, migration, growth, and differentiation [20].

Recently our and other laboratories demonstrated that MSCs isolated from adipose tissue (hADSC) displayed a significant higher proliferative rate and are more potent for lineage-specific differentiation along the three lineages: adipogenic, osteogenic and chondrogenic, compared to bmMSCs [1, 21], thus confirming adipose tissue as the election source to isolate MSCs suitable for cell replacement therapies. Undeniably, hADSCs are either readily available in large quantities and exhibit a very high capability for proliferation and differentiation.

In this work, the osteogenic differentiation potential of hADSCs cultured on an osteo-inductive biomaterial has been evaluated, *in vitro*. For this purpose a collagen/hydroxyapatite scaffold that mimics both the cartilage and bone tissue [18, 22–25] has been used to verify whether hADSCs loaded on scaffold constructs have the potential to differentiate into mature osteoblasts with or without the supplement of differentiation inducing factors.

The results obtained from this study confirmed that hADSCs, expanded *in vitro*, were able to differentiate along the osteogenic way under the stimulus of specific factors. Once loaded on collagen/hydroxyapatite scaffolds, hADSCs were able to differentiate into mature osteoblasts even in absence of specific inducing factors; on the other hand, the presence of these factors was able to trigger an acceleration of the osteo-inductive process, as assessed by the expression of specific markers evaluated by immunohistochemistry, and specific colorimetric assay (Alizarin Red S). Therefore, these results indicate that the biomaterial composing the scaffold is sufficient *per se* to stimulate the host populating cells to differentiate into mature osteoblasts; nonetheless, the presence of osteo-inductive factors can markedly accelerate this process. This observation may have a considerable clinical impact, supporting the potential use of hADSC–scaffold combinations to treat bone diseases and trauma, in those special cases (elderly people, patients with dysfunctions or post-chemotherapy patients) where the physiological osteogenic process is significantly depressed.

## Materials and Methods

### Isolation, expansion and characterization of adipose derived stem cells

hADSCs were derived from adipose tissue biopsies/lipoaspirates supplied by Mediterranean Institute of Oncology (IOM) (Viagrande, Italy) under an approved Institutional Review Board protocol (project ID code: 829_1 of 8 February 2013, IOM Institutional Review Board). Written informed consent has been obtained from all donor patients who agreed to provide samples for the present study. Isolation of hADSCs from adipose tissue was performed as previously reported [1, 26–27]. After isolation, cells were characterized by immunocytochemistry and flow cytometry analysis using several positive (CD105, CD90, CD73) and negative (CD45, CD34 and CD31) mesenchymal stem cells surface markers as previously reported [1, 28].

### hADSCs osteogenic differentiation

For the induction of osteogenic differentiation, hADSCs were seeded at a density of 3,1x10^3^ cells/cm^2^ on collagen I (Serva, Heidelberg, Germany) coated culture slides in expansion medium (ADSC basal medium; Lonza Group: catalog no. PT-3273) supplemented with ADSC-GM SQ (10% FBS and gentamicin-amphotericin B; Lonza Group: catalog no. PT-4503) at 37°C. After 24 hours, the medium was completely replaced with MSC-GM medium supplemented with osteogenic differentiation promoting factors (hMSC osteogenic differentiation BulletKit, Lonza, Basel, Switzerland).

### Gene expression analysis

To confirm the osteogenic induction, total RNA from differentiated hADSCs (d0-d24) was isolated daily through RNeasy minikit (Qiagen, Germantown, MD, USA) according to the manufacturer's instructions. Three independently isolated and cultured samples were used for each of the 25 time points in osteogenic differentiation. cDNA was synthesized from 1μg of total RNA using M-MLV Reverse Transcriptase (Life Technologies, Monza, Italia). Quantitative PCR was performed using SYBR Green method on a 7900HT Real Time PCR (Applied Biosystems). Specific primers for each of the investigated molecular endpoint were designed using primer blast [29] and selecting exon-exon junctions on mRNA as target region for annealing. Each sample was tested in triplicate and gene expression was assessed using the 2^-ΔΔCt^ method as previously described [30]. RNA from undifferentiated hADSCs (defined as cells cultured in expansion medium before the osteogenic induction) were used as reference for relative quantitation. The following primers for real-time PCR were used: Osteocalcin (BGLAP), Osteopontin (SPP1), Alkaline Phosphatase (ALPL), Osterix (SP7), Bone Morphogenetic Protein 2 (BMP2), Cell Division Cycle 25A (CDC25A), Collagen type 1 alpha1 (COL1A1), Collagen type 2 alpha1 (COL2A1), Insulin-like growth factor 2 (IGF2), Integrin, alpha 1 (ITGA1), msh homeobox 1 (MSX1), Runt-related transcription factor 2 (RUNX2), Sex Determining Region Y box 9 (SOX9), Transforming Growth Factor Beta 1 (TGFb1), Vascular Cell Adhesion Protein 1 (VCAM). Oligonucleotide sequences are reported in Table 1. Results were normalized to the levels of Glyceraldehyde 3-Phosphate Dehydrogenase (GAPDH) and Beta-Tubulin (TUBB).

**Table 1:** qRT-PCR primer sequences.

target	forward	reverse	target	forward	reverse
**BGLAP**	GGCAGCGAGGTAGTGAAGAG	GATGTGGTCAGCCAACTCGT	**SPP1**	AGTTTCGCAGACCTGACATCCAGT	TTCATAACTGTCCTTCCCACGGCT
**ALPL**	GACCCTTGACCCCCACAAT	CGCCTCGTACTGCATGTCCCCT	**SP7**	TGCTTGAGGAGGAAGTTCACTATG	TGCCCAGAGTTGTTGAGTCC
**BMP2**	GGAACGGACATTCGGTCCTT	CACCATGGTCGACCTTTAGGA	**CDC25A**	GGCAAGCGTGTCATTGTTGT	GGCTCACAGTAAGACTGGCA
**COL1A1**	CCGGAAACAGACAAGCAACCCAAA	AAAGGAGCAGAAAGGGCAGCATTG	**COL2A1**	TGGTCTTGGTGGAAACTTTGCTGC	AGGTTCACCAGGTTCACCAGGATT
**IGF2**	GCAGAGAGACACCAATGGGAAT	CGGCAGTTTTGCTCACTTCC	**ITGA1**	CCGGTGGAAGACATGTTTGG	GCTGCATTCTTGTACAGGGG
**MSX1**	CCACTCGGTGTCAAAGTGGA	GAAGGGGACACTTTGGGCTT	**RUNX2**	GGAGTGGACGAGGCAAGAGTTT	AGCTTCTGTCTGTGCCTTCTGG
**SOX9**	AACAACCCGTCTACACACAGCTCA	TGGGTAATGCGCTTGGATAGGTCA	**TGFb1**	TGGCGATACCTCAGCAACC	CTCGTGGATCCACTTCCAG
**VCAM**	TGTTTGCAGCTTCTCAAGCTTTTA	TCGTCACCTTCCCATTCAGT	**GAPDH**	GCTCTCCAGAACATCATCCCTGCC	GCGTTGTCATACCAGGAAATGAGCTT
**TUBB**	GCGCATTCCAACCTTCCAG	CCCAGAACTTGGCACCGAT			

### Statistical analysis

For the transcriptional profiling of hADSC during differentiation each investigated marker has been evaluated in six groups of samples (days 0–3, 4–7, 8–11, 12–15, 16–19, 20–24), which were classified according to differentiation time, and in hADSCs in expansion medium (control group). Since three independent cultures have been used for each time points, each group consisted of 12 samples except group 20–24, which consisted of 15 samples (because comprised 5 different time points instead of 4), and reference group, which consisted of 3 samples characterized during expansion. The significance of expression differences has been assessed by Analysis of Variance (AOV) method. Tukey's ‘Honest Significant Difference’ method has been used as post hoc test when the AOV reported statistically significant mRNA modulation in order to identify which time groups were significantly different from the control group.

Differences in cell counts (H&E and immunohistochemistry) and alizarin red staining in temporal groups of samples cultured in both expansion and osteogenic medium has been evaluated using two way ANOVA. Tukey's ‘Honest Significant Difference’ method has been used as post hoc test.

Cell count analysis has been performed using Fiji image recognition software. Optical density measurements have been made using ImageJ software.

### Scaffolds structure

The scaffold was developed through a bio-inspired process, integrating an organic compound (type I collagen) with bioactive Mg-doped hydroxyapatite (Mg-HA) nano-crystals and stabilizing the overall structure by a highly reactive bis-epoxy (1,4-butanediol diglycidyl ether, BDDGE).

The manufacturing process leads to a very good integration between collagen (atelo-collagen isolated from equine tendon) and co-precipitated Mg-HA nanoparticles. A solution prepared with H_3_PO_4_ and highly purified water was mixed with 1wt% type I collagen gel and dropped into basic suspension containing Ca(OH)_2_, MgCl_2_∙6H_2_O and SBF to yield a composite Mg-HA/Collagen material in the theoretical ratio of 70/30wt% and Mg/Ca mol = 5% in the crystal lattice. The precipitated fibers were matured for 1hr and subsequently washed with highly purified water.

In order to optimize the molecular links between collagen and BDDGE, the biomineralized collagen was chemically crosslinked by 48hrs-long immersion at 37°C and pH = 9.5 in NaHCO_3_/Na_2_CO_3_ buffered aqueous solution with a BDDGE/volume solution ratio equals to 1wt%. Finally, in order to consolidate the 3D structure each layer was overlapped and the whole structure was freeze-dried with a controlled freezing and heating ramp from 25°C to -35°C and from -35°C to 25°C. The process was carried out over a period of 25 hours under vacuum conditions (P = 0.29 mbar) and then samples were gamma-sterilized at 25kGy.

The morphological and microstructural analysis of the bone-like materials was executed by Scanning Electron Microscopy (SEM) performed on a SEM-LEO 438 VP (Carl Zeiss AG, Oberkochen, Germany). The samples were sputter-coated with gold prior to examination.

In particular, the SEM analysis has been carried out on 7 SEM micrographs analyzed by image J software. The mean pore diameter was calculated as average of the major and minor axes of the ellipse representing pores cross-section. A total of 134 pores were analyzed obtaining a mean value of 78±28 micron. A quantitative analysis of pore size distribution shows that the 50% of pores are between 50 and 80 micron. Sporadic large channels (300–500 micron) are visible in the microstructure but they have been excluded from the analysis.

Analysis of the structural stability was performed in triplicate on crosslinked and non-crosslinked samples, monitoring the effects of the crosslinker in carbonate solution at 37°C. Degradation rate of samples treated with collagenase (100u/ml) was defined monitoring the samples (10mg) by visual inspection every 30 minutes up to the complete degradation. Data have been analyzed using t student to highlight statistical difference on stability between the two groups (p<0.05).

The specific scaffold composition was verified by Thermogravimetric Analysis (TGA), carried out with a Mettler Toledo DT-TGA/DSC1 Star System (Columbus, OH, USA). Analysis was performed in triplicate inside alumina crucibles in air atmosphere with a flow rate of 80 ml/min, in a temperature interval between 40 and 1000°C. The percentage of the inorganic mineral content has been calculated as the residual mass at the end of the TGA analysis respect to the initial one. Samples have been tested in triplicate.

### hADSC osteogenic differentiation on scaffolds

2x10^6^ hADSCs at passage 3 were slowly drip seeded onto the bone single-layer scaffold and incubated in 24-well culture plates for 4 hours at 37°C. ADSC-GM medium (2ml) (Lonza, Basel, Switzerland) was added and 24hr later (day0) the medium was replaced with osteogenic medium. The osteogenic medium was completely replaced twice a week. Each scaffold was analysed on day 7, 14, 28 and 56, after osteogenic induction.

### Immunoistochemistry analysis on hADSCs-scaffold

Scaffolds seeded with hADSC were fixed in 4% PFA, dehydrated, embedded in paraffin and cut into 3 μm-thick sections. Sections were mounted on slides and processed for immunohistochemical staining. After deparaffinization and rehydration, sections were permeabilized with 0.4% Triton-X100, blocked with 4% BSA and then incubated overnight at 4°C with one of the following rabbit polyclonal primary antibodies: anti-osteopontin (SPP1; 1:250, Novus Biological, Cambridge, UK), anti-osteocalcin (BGLAP; 1:50, LSBio, Seattle, WA, USA), anti-alkaline phosphatase (ALPL; 1:50, LSBio), anti-osterix (SP7; 1:250, Biorbyt, Cambridge, UK) and anti-osteonectin (SPARC; 1:50, LSBio). The following day, sections were incubated for 1 h at RT with the Alexa Fluor anti-rabbit 568 secondary antibodies (1:2000, Life Technologies Italia, Monza, Italy). Then slides were counterstained with 4',6-diamidino-2-phenylindole (DAPI) (1:10.000) and mounted with Permafluor (Thermo Scientific, Waltham, Massachusetts, USA). Control of immunostaining specificity was performed omitting the primary antibody. Alternate sections were also stained with either haematoxylin and eosin (H&E) or Alizarin Red S (Panreac, Castellar del Valles, Barcellona, Spain). For the staining, the Alizarin Red S solution was prepared according to the manufacturer protocol and sections were incubated for 5 minutes, washed several times to get rid of the excess of the staining solution and, finally, mounted.

## Results

### hADSCs osteogenic differentiation and gene expression profiling

The osteogenic potential of hADSCs cultured on collagen I coated plastic dishes, in presence of differentiation inducing factors, has been extensively characterized. For this purpose, hADSCs were isolated from three different adipose tissue/lipoaspirate samples by adhesion to plastic dishes and independently characterized by flow cytometry using several mesenchymal stem cells surface markers which expressions are reported below as mean±standard error. Our results consistently indicate a very low expression of differentiation markers such as CD45 (2.9%±2.1), CD34 (3.3%±1.9) and CD31 (2.2%±0.9), and high levels for stem cells specific markers CD271 (89.2±5.7), CD105 (88.4%±4.0), CD90 (87.0%±6.2) and CD73 (95.1%±1.7).

Cells were then plated in presence of specific osteogenic factors for 24 days. During hADSCs in vitro osteogenic differentiation, the expression level of several transcription factors and bone related genes has been measured by Real Time qRT-PCR on each of the 24 days of induction. The assessed molecular endpoints are functionally related to skeletal development (BMP2, COL1A1, COL2A1, IGF2, MSX1, SP7, SPP1), bone mineralization (BGLAP), cartilage condensation (SOX9), ossification (ALPL, BGLAP, RUNX2), osteoclast differentiation (BGLAP), bone mineral metabolism (BGLAP, COL1A1, COL2A1), cell growth and differentiation (CDC25A, IGF2, TGFβ1, BMP2), extracellular matrix maturation (COL1A1, COL2A1, BGLAP, BMP2, IGF2), cellular adhesion (VCAM1, ITGA1, BGLAP) and regulation of gene expression during bone formation (MSX1, RUNX2, SOX9, SP7) [31–34].

**Fig 1:**
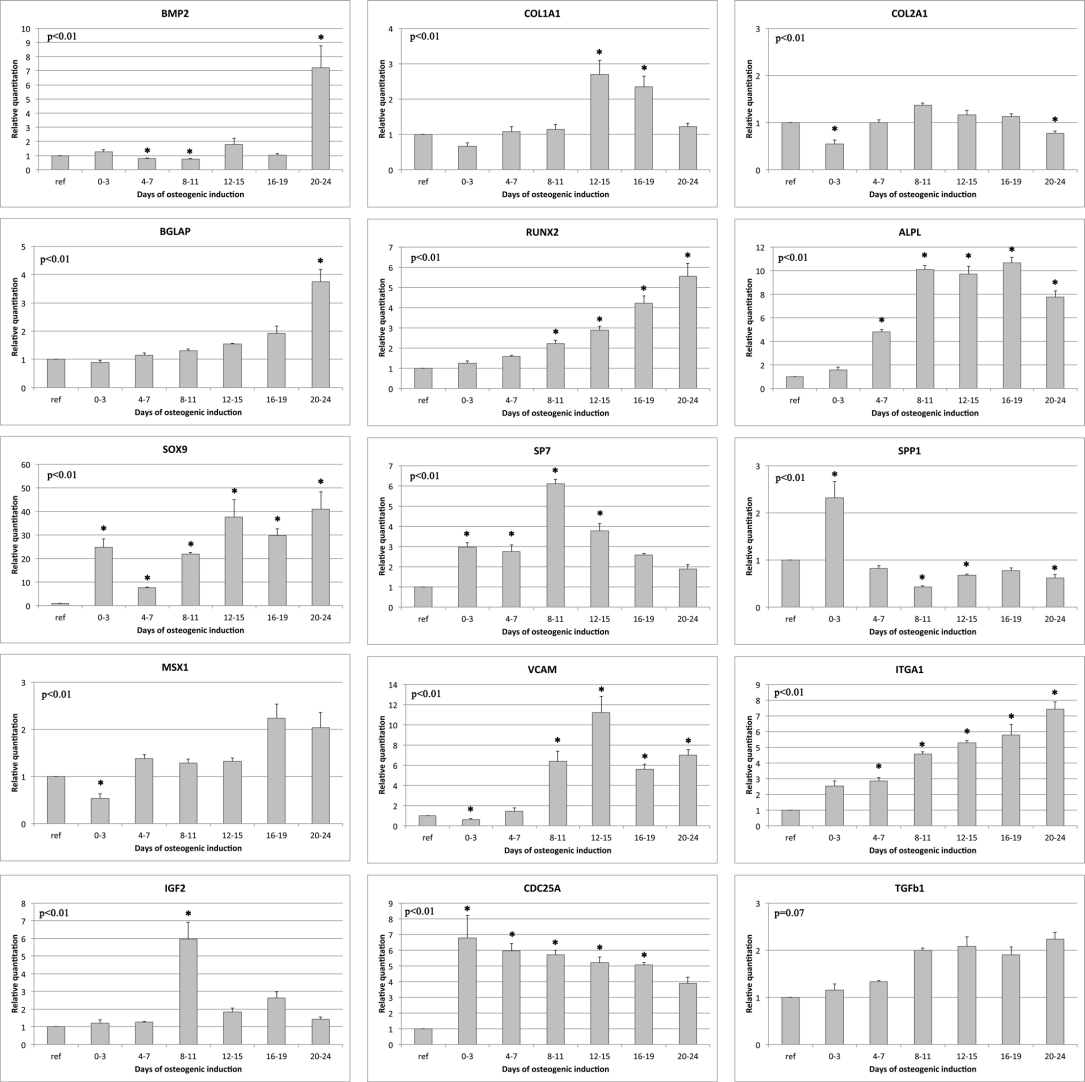
Gene expression during hADSCs osteogenic differentiation on plastic dishes. Graphical representation of relative gene expression during 6 temporal intervals (days 0–3, 4–7, 8–11, 12–15, 16–19, 20–24) using hADSC in expansion medium as control group. qRT-PCR has been performed for the mRNA of Osteocalcin (BGLAP), Osteopontin (SPP1), ALPL, Osterix (SP7), BMP2, CDC25A, COL1A1, COL2A2, IGF2, ITGA1, MSX1, RUNX2, SOX9, TGFb1, VCAM. Glyceraldehyde-3-phosphate dehydrogenase (GAPDH) and Beta-Tubulin (TUBB) have been used as endogenous controls. AOV test p-value is reported and * indicates significant (p<0.05) differences between time groups and control samples as reported by the post-hoc test.

Relative quantitation values are reported as measure of gene expression variations for samples grouped in 6 temporal intervals (days 0–3, 4–7, 8–11, 12–15, 16–19, 20–24) (Fig 1).

Early osteogenic markers, like SPP1 (Osteopontin) and ALPL (Alkaline Phosphatase), rapidly increase their expression during the initial phases of differentiation, while late markers, like BGLAP and BMP2 show concentration peaks during intermediate or late stages. The augmented expression of CDC25A and ITGA1 are coherent with improved proliferation and cell cycle progression induced by osteogenesis. Genes involved in extracellular matrix maturation and cellular adhesion are shown to be activated during the last period of osteogenic differentiation.

### Biomimetic scaffolds fabrication and characterization

**Fig 2:**
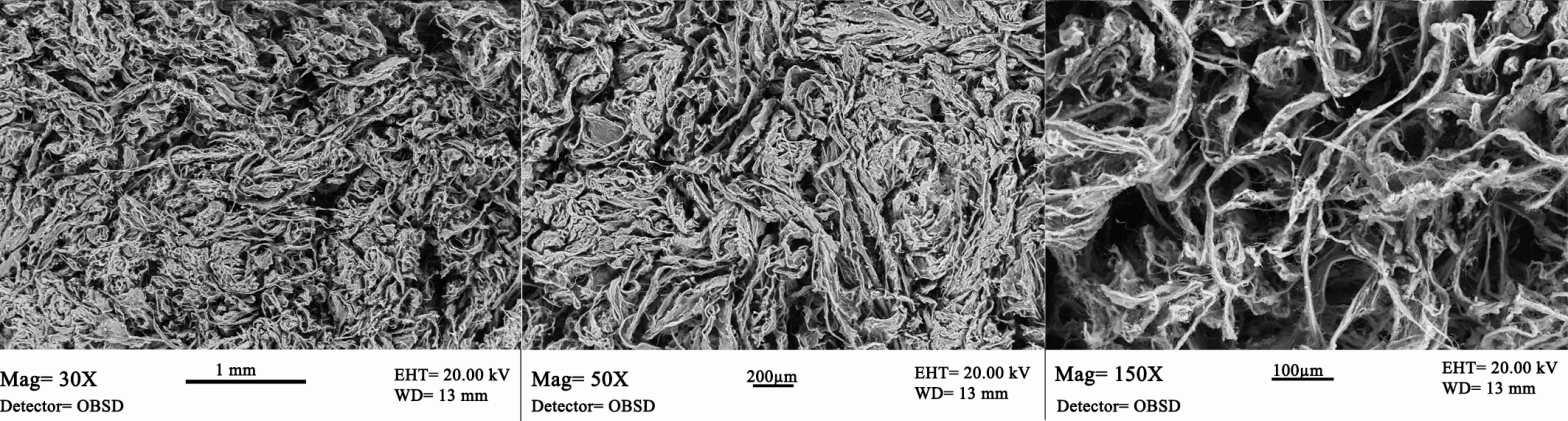
Representative SEM images of the scaffold bony layer. Ultrastructural details of the bony layer are reported with three different magnification 30x, 50x and 150x.

The morphological and microstructural analysis of the scaffold was performed by SEM. Representative SEM images of the bony layer are reported with three different magnification 30x, 50x and 150x (Fig 2).

The bone-like substitute shows well interconnected pores with diameters up to 300 micron and evident large channel around 600 micron. The biomineralized collagen fibres create a highly fibrous structure.

The enzymatic degradation of the bone scaffold layer showed a very slow profile with complete degradation in more than 150 days.

Mineral content of scaffolds produced with different collagen batches at 37°C in buffer carbonate solution with 1% of BDDGE, have been evaluated by biomineralization degree and collagen degradation temperature. Our results indicate a biomineralization degree of 50.5%±1.0 Mg-HA and collagen degradation temperature of 319°C ±3.4.

### hADSC-scaffold biocompatibility

**Fig 3:**
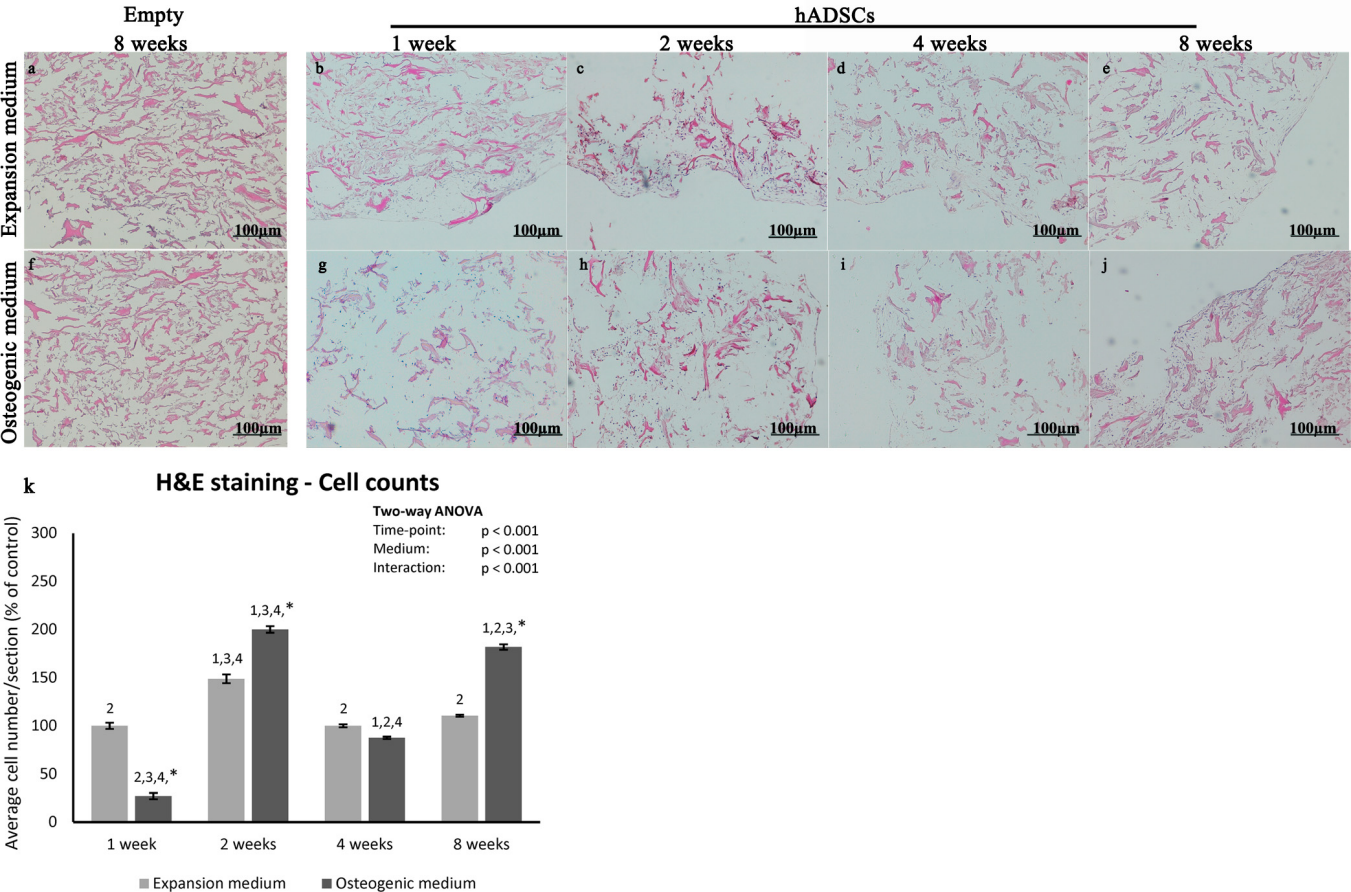
Biocompatibility of biomimetic scaffold. Haematoxylin and eosin staining of empty scaffolds maintained for 8 weeks in expansion (a) or osteogenic medium (f) and of hADSCs seeded scaffolds (b-e, g-l) maintained in the same media for 1, 2, 4 and 8 weeks, magnification 10x. (k) Graphical representation of cell count at 1, 2, 4 and 8 weeks of culture in expansion (light bars) and osteogenic medium (dark bars). Two-way ANOVA p values are reported. Symbols above bars indicate statistically significant differences (p<0.05) in the Tukey HSD post-hoc tests: 1 indicates differences with the 1 week group (same medium), 2 indicates differences with the 2 weeks group (same medium), 3 indicates differences with the 4 weeks group (same medium), 4 indicates differences with the 8 weeks group (same medium) and * indicates differences in osteogenic vs expansion medium groups (same time-points). Three independently isolated hADSC samples have been used for each time-point.

Once assessed the hADSC osteogenic potential, we wanted to evaluate the hADSCs ability to adhere and invade the bone single-layer scaffold. For this reason, hADSCs were loaded on scaffold and maintained in culture during the osteogenic differentiation; afterwards, hADSC-scaffold structures were processed for histological analysis and sections were stained at different time points (1, 2, 4 and 8 weeks) with haematoxylin and eosin (Fig 3).

We found that cells were able to penetrate, adhere and proliferate into the scaffold. In particular, the cell number significantly increased with time and, while at the earlier time points, cells were confined only on the surface of the scaffold, later on cells started to invade also the inner part of the scaffold, penetrating deeply inside. This was observed in both experimental culture conditions: with growth medium (ADSC-GM, Fig 3B–3E) and with osteogenic medium (MSC-GM supplemented with osteogenic differentiation factors, Fig 3G–3J). Nuclei counts reveals that the number of cells invading the scaffolds is significantly higher at 2 and 8 weeks when cells are grown in osteogenic medium (Fig 3K).

### Collagen/hydroxyapatite scaffold induces in vitro hADSC osteogenic differentiation

**Fig 4:**
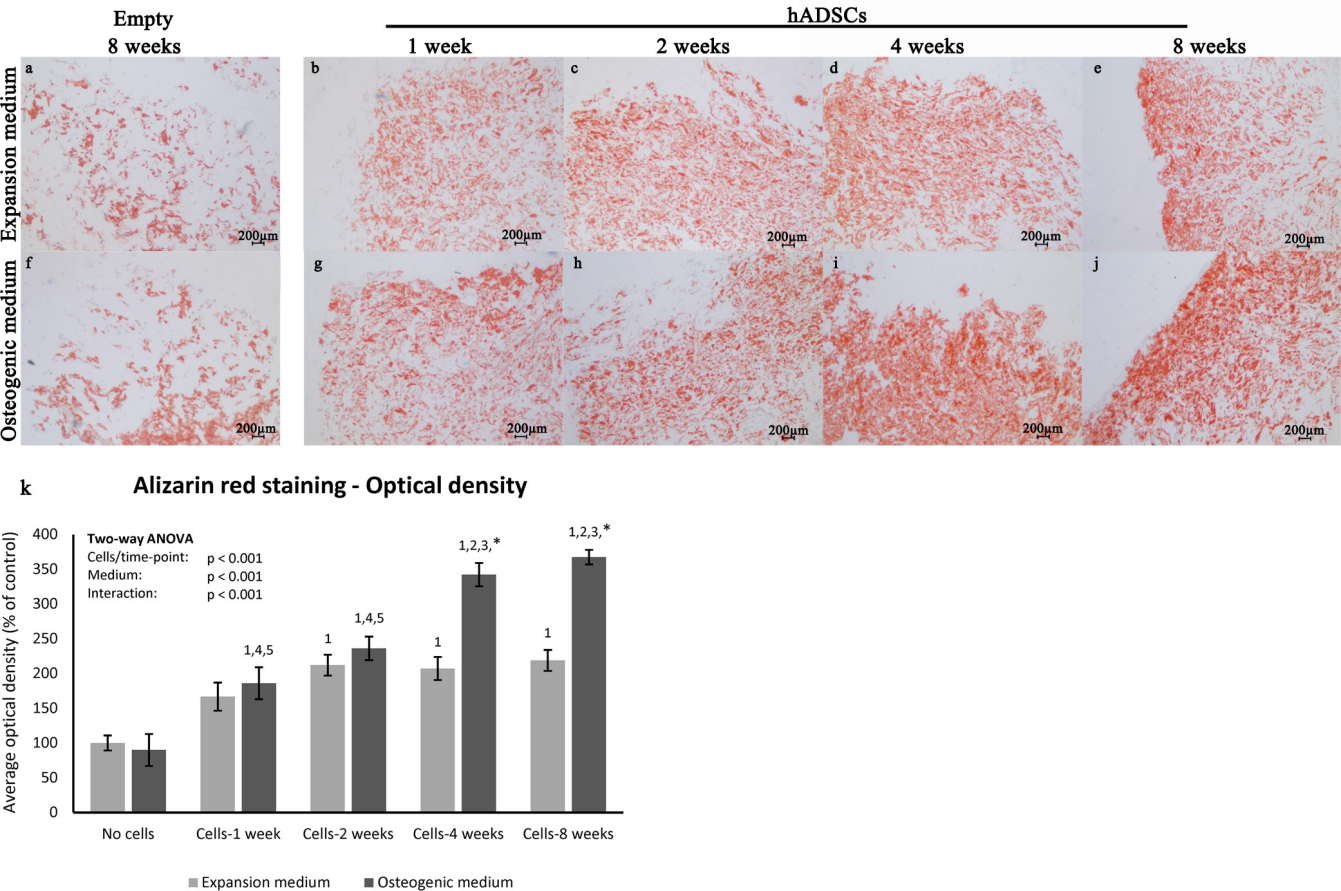
Matrix mineralization during osteogenesis. Alizarin Red S staining of empty scaffolds maintained for 8 weeks in expansion (a) or osteogenic medium (f) and of hADSCs seeded scaffolds (b-e, g-j) maintained in the same media for 1, 2, 4 and 8 weeks, magnification 2.5x. (k) Graphical representation of Alizarin Red S staining resulting optical density at 1, 2, 4 and 8 weeks of culture in expansion (light bars) and osteogenic medium (dark bars) and of empty scaffolds at 8 weeks. Two-way ANOVA p values are reported. Symbols above bars indicate statistically significant differences (p<0.05) in the Tukey HSD post-hoc tests: 1 indicates differences with the empty scaffold group (same medium), 2 indicates differences with the 1 week group (same medium), 3 indicates differences with the 2 weeks group (same medium), 4 indicates differences with the 4 weeks group (same medium), 5 indicates differences with the 8 weeks group (same medium) and * indicates differences in osteogenic vs expansion medium groups (same time-points). Three independently isolated hADSC samples have been used for each time-point.

The osteo-inductive character of the scaffold has been evaluated together with the differentiation potential of hADSCs in both presence and absence of osteogenic inducing factors in culture media. This evaluation was carried out through Alizarin Red S staining and immunohistochemistry analysis performed on hADSC-scaffold sections, at 1, 2, 4 and 8 weeks. The Alizarin Red S staining revealed the presence of calcium stores in hADSCs extracellular matrix, in both culture conditions (Fig 4).

The analysis of Alizarin red staining optical density variation show that mineralization of hADSC loaded scaffolds is significantly higher than the empty scaffolds used as control at any experimental time. Moreover, when samples are cultured in expansion medium, the calcium stores start to appear at 2 weeks and gradually increase up to 8 weeks, where the content of calcium results quite evenly diffused throughout the scaffold (Fig 4B–4E and 4K). On the other hand, when samples are grown in presence of osteogenic factors, the mineralization of the extracellular matrix starts as early as the first week with a statistically significant increase over time as well with a greater amount of calcium deposition at each of the assessed time-points (Fig 4G–4J and 4K) compared to those in expansion medium. The optical density variation indicates also that the above-mentioned differences in matrix mineralization are statistically significant. Hence, these data show that the scaffold *per se* is able to induce hADSC osteogenic differentiation *in vitro*; nevertheless, the presence of osteogenic differentiation promoting factors accelerates and strengthens this process, as resulted from the substantial augmentation of number and size of calcium content in the extracellular matrix.

**Fig 5:**
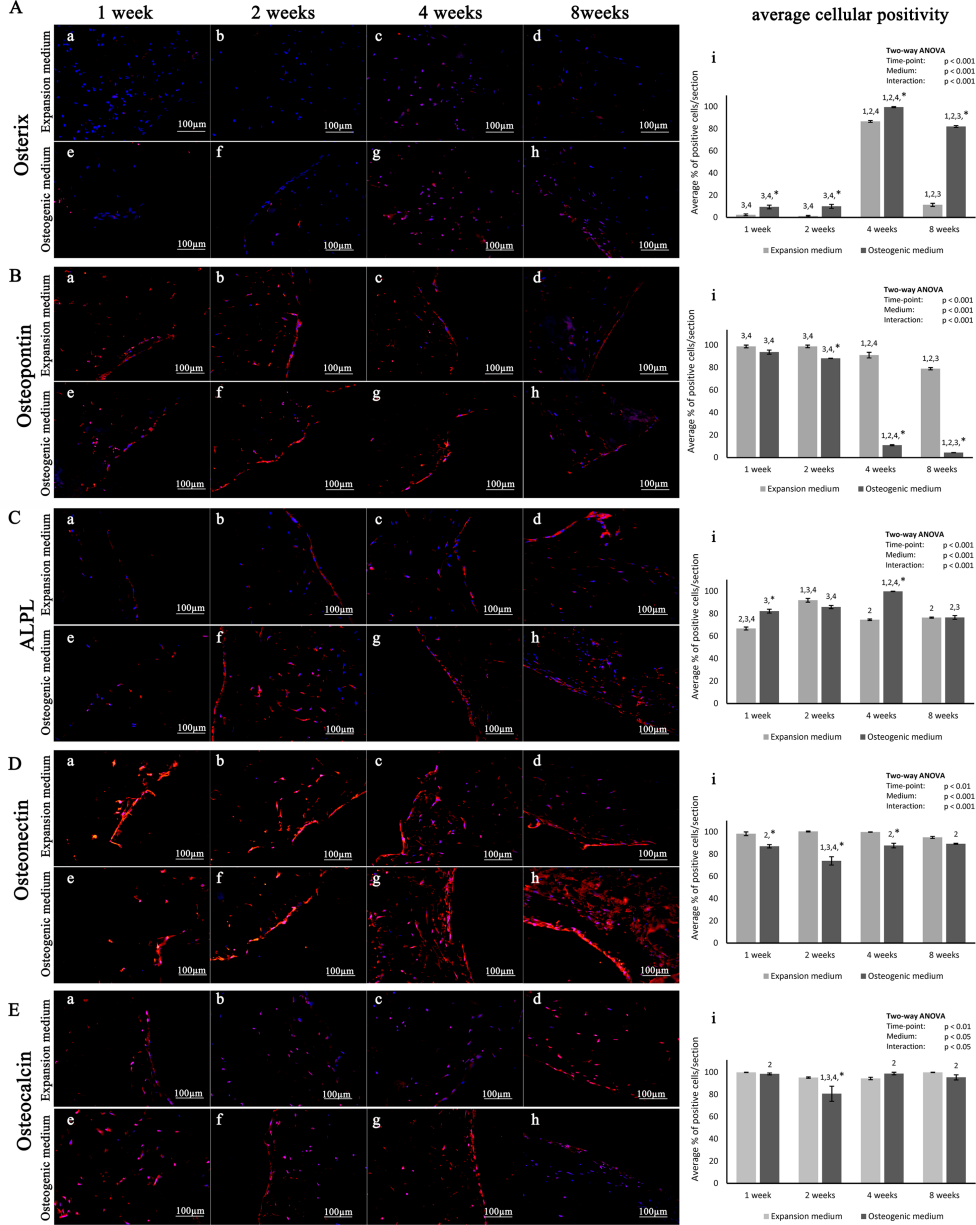
Expression of representative osteogenic markers. Immunohistochemical analysis of representative osteogenic markers: (A) Osterix, (B) Osteopontin, (C) ALPL, (D) Osteonectin and (E) Osteocalcin, performed on hADSCs cultured on scaffolds either in expansion (a-d) or in osteogenic medium (e-h), at different time points (1, 2, 4 and 8 weeks), magnification 20x. (i) Average cellular positivity for osteogenic markers in both expansion (light bars) and osteogenic medium (dark bars). Two-way ANOVA p values are reported. Symbols above bars indicate statistically significant differences (p<0.05) in the Tukey HSD post-hoc tests: 1 indicates differences with the 1 week group (same medium), 2 indicates differences with the 2 weeks group (same medium), 3 indicates differences with the 4 weeks group (same medium), 4 indicates differences with the 8 weeks group (same medium) and * indicates differences in osteogenic vs expansion medium groups (same time-points). Three independently isolated hADSC samples have been used for each time-point.

Moreover, the scaffold osteo-inductive character on hADSC was also evaluated by immunohistochemistry using several specific markers associated with different stages of osteogenic differentiation. Statistical analysis of the average cellular positivity for each of the investigated markers indicates that all the assessed molecular endpoints are significantly modulated during osteogenesis. Details on specific variations are reported in Fig 5.

Immunohistochemical analysis for Osterix, a transcription factor specifically expressed by osteoblasts, shows a positive staining in hADSCs, both in absence and in presence of osteogenic differentiation medium, although with different intensity at the same time points.

In particular, cells in expansion medium start to show an Osterix signal at 4 weeks, corresponding to its highest expression. In fact, the protein levels are already reduced at 8 weeks (Fig 5Aa-d). Instead, in osteogenic differentiation medium, Osterix expression starts to be weakly visible after 2 weeks. Even in this case the expression reaches a peak at 4 weeks and then clearly decrease at 8 weeks (Fig 5Ae-h). However, at the same time points, cells in differentiation medium express Osterix in higher percentage and with greater intensity then those in expansion medium.

Osteopontin, a typical marker of pre-osteoblast and mature osteoblast stages, shows a positive signal in each time point of the two different culture conditions. In both cases, there is a peak of expression at 2 weeks and, afterwards, the signal is slightly reduced up to 8 weeks (Fig 5Ba-d and 5Be-h). The main difference between the two conditions is that cells grown in osteogenic medium express higher levels of Osteopontin at each analysed time point.

Alkaline Phosphatase, a marker associated with matrix maturation, exhibits a basal signal since the first week with expression gradually increasing up to 8 weeks. Even in this case, cells growth in osteogenic medium exhibit higher levels of ALPL at each time point analysed (Fig 5Ca-h).

Immunohistochemical analysis of Osteonectin (SPARC), a specific marker associated with bone mineralization stage, reveals a strong expression of this marker since the first week, in both cell culture conditions. However, the expression levels seem unchanged along the time points in expansion medium, whereas they increase markedly at the later stages of differentiation in the presence of osteo-inducing factors (3 and 4 weeks) (Fig 5Da-h).

The expression of Osteocalcin, a specific marker of mature osteoblasts, is already detectable at 1 week in both experimental conditions (Fig 5E). However, it appears unaltered during the first four weeks and increased at 8 weeks (Fig 5Ea-d) when cultured in expansion medium. On the contrary, cells cultured in osteogenic medium show a stronger signal during the first four weeks, followed by an evident reduction at eight weeks (Fig 5Ee-h). In this case, the presence of the osteogenic factors probably accelerates the differentiation process resulting in an earlier appearance of the marker.

## Discussion

We have recently shown that MSCs isolated from adipose tissue exhibited a higher proliferation and differentiation potential along adipogenic, osteogenic and chondrogenic lineages, compared to bmMSCs [1], thus suggesting the use of this cell line for regenerative medicine. In this work, we first performed a gene expression analysis of mRNAs involved in osteogenic differentiation as a molecular readout for the process and then assessed the osteogenic differentiation potential of hADSCs once loaded on an osteo-inductive biomaterial, *in vitro*.

To this aim, we evaluated changes in gene expression profile in hADSCs during the different stages of osteogenic differentiation. Several transcription factors and genes involved in bone formation were studied by real time qPCR during 24 days of the osteogenic induction. In particular, we focused our attention on genes involved in skeletal development, bone mineralization, cartilage condensation, ossification, osteoclast differentiation, bone mineral metabolism, cell growth and differentiation, extracellular matrix maturation, cellular adhesion and regulation of gene expression in bone formation.

MSX1, SOX9, RUNX2 and SP7 are the major transcriptional controllers of proliferation and differentiation of MSCs into mature bone cells. Our results demonstrate that RUNX2 and SP7, which are positive transcriptional regulators of genes like type I collagen, osteopontin, bone sialoprotein, osteocalcin and osteonectin [35–37], are positively modulated during osteogenesis with a distinctive expression profile. While RUNX2 exhibits a linear increase in mRNA levels during differentiation, SP7 levels increase more rapidly with a peak of transcriptional activity during days 8–11 and a decrease during the stages of matrix mineralization. The complex interplay between RUNX2 and SP7 is clearly reflected on the modulation of target genes during different phases of differentiation. While SP7 activation during osteoblast formation contributes to SPP1 early activation, its gradual decrease after day 11 is known to be required to promote osteoblast differentiation at late stage in a RUNX2 independent manner [38]. On the other hand, the gradual increase of RUNX2 is coherent with its role in the temporal activation of Col1A1, ALPL and BGLAP, which are involved in intermediate and later stages of matrix maturation and bone mineralization.

Members of the transforming growth factor (TGF)-β family including BMP2, proteins such as ALPL, COL1A1, COL2A1, BGLAP, SPP1 and factors like CDC25A, IGF2, ITGA1 and VCAM play a role in cell adhesion, proliferation, extracellular matrix maturation and differentiation of the osteoblast phenotype [39–43]. As clearly indicated by activation trends of these genes, cell proliferation and progression through cell cycle are quickly and greatly promoted by induction since the initial stages of osteogenic differentiation. Moreover, the distinctive temporal regulation of IGF2, that exhibits a conspicuous increase in mRNA levels during day 8–11, suggests that its activation is functionally linked to ALPL and other late markers transcriptional activation in agreement with previously reported evidences [44].

Once assessed the molecular profile of hADSCs during lineage commitment and bone differentiation, we evaluated the osteogenic differentiation potential of this cells layered on the top of a scaffold and maintained in culture. A scaffold is a three-dimensional structure capable to support cell colonization, proliferation, and differentiation of appropriate cells [45]. Several types of scaffold, either of synthetic and biological formulation, have been already used to support osteogenic differentiation of MSCs [46–48]. Stimuli mimicking the *in vivo* bone environment are needed for encouraging cells to differentiate into mature osteoblasts forming new bone tissue. In our model, the scaffold is a combination of collagen and Mg-hydroxyapatite (HA). The data obtained from our study showed that the scaffold *per se* is sufficient to stimulate *in vitro* osteogenic differentiation of hADSCs layered on top. This is in line with data reported in literature, in fact, collagen is the main component of bone tissue, where it stimulates MSCs to differentiate into osteoblasts, initiating new bone formation [45]. Moreover, it has been already shown that scaffolds containing HA improved cell proliferation and promoted production of mineralized extracellular matrix (ECM) more than that observed for the scaffold without HA [49]. Nonetheless, the addition of osteo-inductive factors to the culture medium significantly accelerated the osteogenic process, already stimulated by the collagen and HA composing the scaffold. This is suggested by the up-regulation and earlier appearance of osteogenic markers, and by the faster and more consistent mineralization of the ECM. The former is supported by immunohistochemical analysis of lineage specific markers for osteoblastic differentiation (Fig 5), while the latter is clearly suggested by increased Alizarin Red S staining when cells are subjected to osteo-inductive soluble factors (Fig 4).

Observed timing in SPP1 expression, which is essential for bone formation, is in agreement with previously reported evidences [50]. This protein is consistently produced during the whole period (from 1 week to 8 weeks) with a peak of expression at 4 weeks. SPP1 is synthesized by preosteoblasts, osteoblasts and osteocytes, is secreted into osteoid and is incorporated into bone. Another typical marker of osteoprogenitor cells is BGLAP that is mainly expressed in the latest stages of differentiation (8 weeks) during pre-osteoblast and mature osteoblast transition [51]. Our data indicates that the osteo-inductive factors, added in growth medium, greatly support the osteogenic differentiation process producing an earlier BGLAP appearance. Furthermore, our results support the important role of Osteonectin in linking the bone mineral and collagen phases, probably initiating active mineralization in normal skeletal tissue [52]. In fact, its expression is visible starting from the early stages and maintains levels of expression almost unchanged up to the later stages of differentiation.

SP7 protein expression profiling indicates that this transcription factor starts to accumulate in the nucleus from the fourth week until the end of osteogenic differentiation in agreement with its role of ECM protein regulator [53]. SP7 controls the expression of proteins involved in terminal osteoblast differentiation. In agreement with recent literature [54] that indicates ALPL as a essential factor for mineralization of osteoblastic cells, or data show that ALPL protein expression start to be evident at 2 weeks and increase in the later stages.

The reported evidences clearly demonstrate that the osteo-inductive features of this collagen-hydroxyapatite scaffold support the potential of hADSCs inducing the formation of new bone tissue; thus suggesting that this combination may represent a suitable tool to promote the migration, proliferation, and differentiation of bone cells enhancing regeneration and fracture healing.

## Supporting Information

S1 Manuscript Data Set Complete data set. (XLS)
